# Drp1-dependent mitophagy protects against cisplatin-induced apoptosis of renal tubular epithelial cells by improving mitochondrial function

**DOI:** 10.18632/oncotarget.15470

**Published:** 2017-02-18

**Authors:** Chuanyan Zhao, Zhuyun Chen, Jia Qi, Suyan Duan, Zhimin Huang, Chengning Zhang, Lin Wu, Ming Zeng, Bo Zhang, Ningning Wang, Huijuan Mao, Aihua Zhang, Changying Xing, Yanggang Yuan

**Affiliations:** ^1^ Department of Nephrology, the First Affiliated Hospital of Nanjing Medical University, Nanjing Medical University, Nanjing, China; ^2^ Department of Pharmacy, Xinhua Hospital Affiliated to Shanghai Jiaotong University School of Medicine, Shanghai, China; ^3^ Department of Nephrology, Nanjing Children's Hospital, Nanjing Medical University, Nanjing, China; ^4^ Institute of Pediatrics, Nanjing Medical University, Nanjing, China

**Keywords:** Drp1, mitophagy, mitochondrial dysfunction, mitochondrial fission, cisplatin

## Abstract

Cisplatin chemotherapy often causes acute kidney injury (AKI) in cancer patients. There is increasing evidence that mitochondrial dysfunction plays an important role in cisplatin-induced nephrotoxicity. Degradation of damaged mitochondria is carried out by mitophagy. Although mitophagy is considered of particular importance in protecting against AKI, little is known of the precise role of mitophagy and its molecular mechanisms during cisplatin-induced nephrotoxicity. Also, evidence that activation of mitophagy improved mitochondrial function is lacking. Furthermore, several evidences have shown that mitochondrial fission coordinates with mitophagy. The aim of this study was to investigate whether activation of mitophagy protects against mitochondrial dysfunction and renal proximal tubular cells injury during cisplatin treatment. The effect of mitochondrial fission on mitophagy was also investigated. In cultured human renal proximal tubular cells, we observed that 3-methyladenine, a pharmacological inhibitor of autophagy, blocked mitophagy and exacerbated cisplatin-induced mitochondrial dysfunction and cells injury. In contrast, autophagy activator rapamycin enhanced mitophagy and protected against the harmful effects of cisplatin on mitochondrial function and cells viability. Suppression of mitochondrial fission by knockdown of its main regulator dynamin-related protein-1 (Drp1) decreased cisplatin-induced mitophagy. Meanwhile, Drp1 suppression protected against cisplatin-induced cells injury by inhibiting mitochondrial dysfunction. Our results provide evidence that Drp1-depedent mitophagy has potential as renoprotective targets for the treatment of cisplatin-induced AKI.

## INTRODUCTION

Cisplatin is a widely used chemotherapeutic in the treatment of various solid tumors. However, acute kidney injury (AKI) is the most critical dose-limiting toxicity in cancer patients treated with cisplatin. Although the exact mechanism underlying cisplatin nephrotoxicity is not clear, proximal tubule cell death is recognized as a notable pathological feature. Central to tubular injury is mitochondrial dysfunction [1]. Mitochondrial damage has been observed in several models of cisplatin induced nephrotoxicity and neurotoxicity [2]. Recent report signified that cisplatin accumulated in mitochondria and caused mitochondrial dysfunction, finally resulting in proximal tubular cells death [3]. The manipulation of mitochondrial function may provide a novel therapeutic option to minimize cisplatin nephrotoxicity.

Autophagy is the process responsible for recycling organelles and long-lived proteins to maintain cellular homeostasis [4]. Light chain 3 (LC3) is one well-studied marker of autophagy. Cytosolic LC3-I is converted to the second form LC3-II when autophagy occurs [5]. The ubiquitin-associated protein p62 can also be used to measure autophagy. P62 is recruited to the autophagosomal membrane through interaction with LC3 and is efficiently degraded by autophagy [6]. Targeted degradation of mitochondria by autophagic machinery is referred to as mitophagy. Mitophagy selectively removes dysfunctional or damaged mitochondria and maintains healthy mitochondria population [7]. Mitophagy is an important mitochondrial quality control mechanism [8]. Timely removal of the damaged mitochondria by mitophagy could avoid the consequences of mitochondria-mediated injury [9]. Previous study demonstrating that autophagy eliminates ROS-producing damaged mitochondria presumably through mitophagy during cisplatin nephrotoxicity [10]. However, the precise role of mitophagy and its molecular mechanisms in the pathophysiology of AKI remain unclear.

Several evidences have shown that mitochondrial fission coordinates with mitophagy. It is generally accepted that mitochondrial fission is a pre-requisite for mitophagy in many mammalian cell types. The main regulator of mitochondrial fission is a member of the dynamin family of GTPases named dynamin-related protein-1 (Drp1). Drp1 is a cytosolic protein that translocates at the mitochondrial outer membrane to initiate the fission process. In the absence of mitochondrial division mediated by Drp1, mitochondria showed increased connectivity and size and became defective in mitophagy in mouse heart and brain [11]. Although it was reported that Drp1-mediated mitochondrial fragmentation contributes to cisplatin-induced nephrotoxicity [12], the role of mitochondrial fission on mitophagy remains to be determined.

In the present study, we will determine if mitophagy is protective against cisplatin-induced renal tubular epithelial cells injury via improving mitochondrial function. We also assessed whether mitophagy was triggered by drp1-mediated mitochondrial fission. These results indicate that the key components of mitochondrial quality control are potential therapeutic targets for the treatment of AKI.

## RESULTS

### Cisplatin induced autophagy and mitophagy in HK2 cells

**Figure 1:**
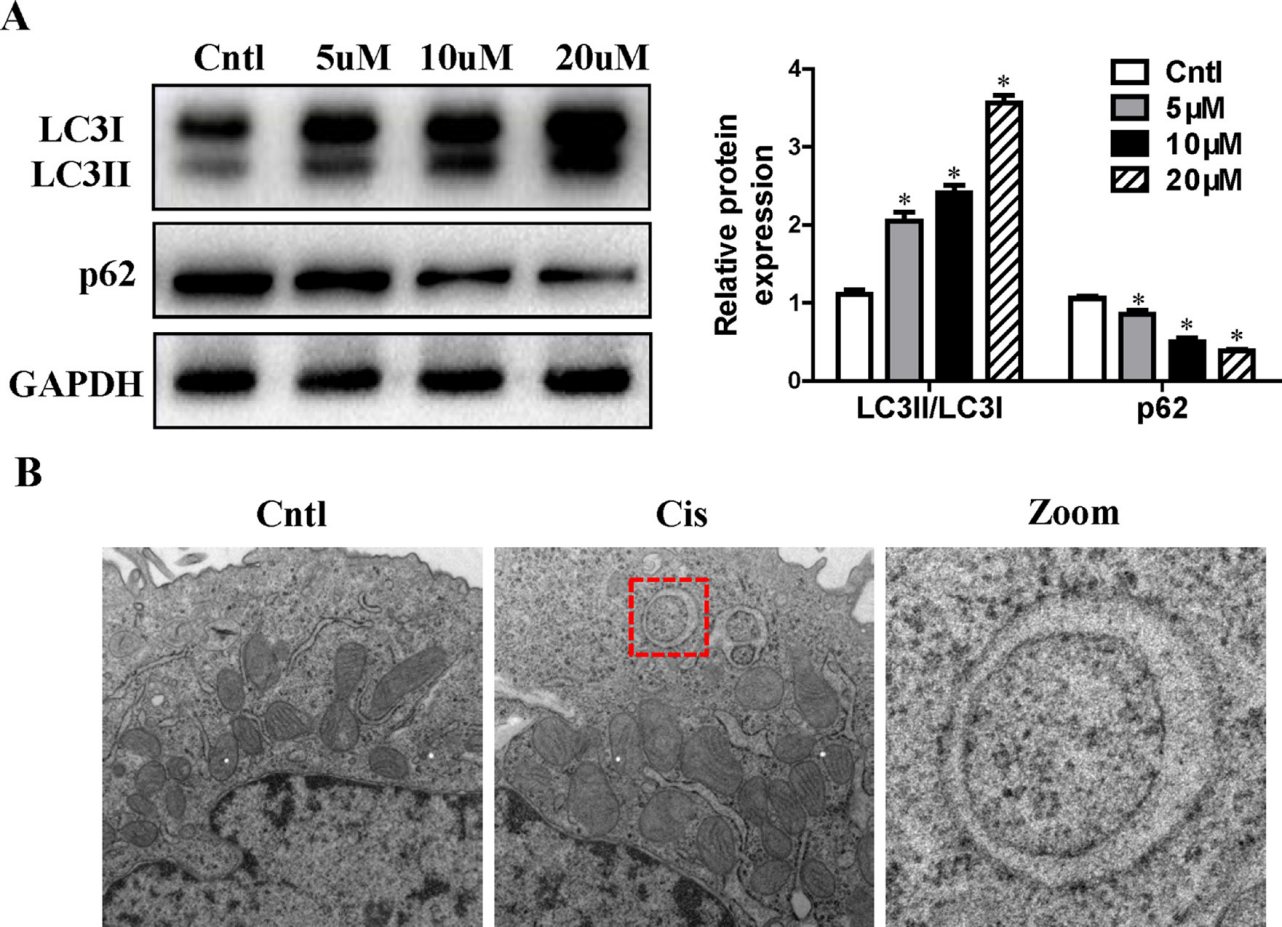
Effect of cisplatin on autophagy in HK2 cells (**A**) HK2 cells were treated with cisplatin at indicated doses for 12 h. LC3 conversion and p62 were detected by immunoblotting. Left: representative immunoblots. Right: densitometric analysis. (**B**) Confluent HK2 cells were incubated with 10 uM cisplatin for 12 h. Representative electron micrographs of autophagosomes engulfing mitochondria in HK2 cells were shown (×20,000). Images at higher magnification are shown in the right panels. Values are means ± SEM from three independent experiments. *P < 0.01 versus control group. Cntl, control group; Cis, cisplatin group.

Previous study demonstrated that autophagy induction was an immediate response of renal tubular epithelial cell exposure to cisplatin [13]. As expected, western blot analysis showed that cisplatin significantly induced the conversion of LC3-I to LC3-II and decreased p62 expression in a dose-dependent manner (Figure 1A). We further verified cisplatin-induced mitophagy in HK2 cells by electron microscopy. As shown by representative micrographs, autophagosomes with characteristic double membranecoated vesicle were identified in the cells after 12 h of cisplatin treatment (Figure 1B). These results indicate the presence of active mitophagy after cisplatin treatment.

### Autophagy inhibitor prevented cisplatin-induced mitophagy in HK2 cells

**Figure 2:**
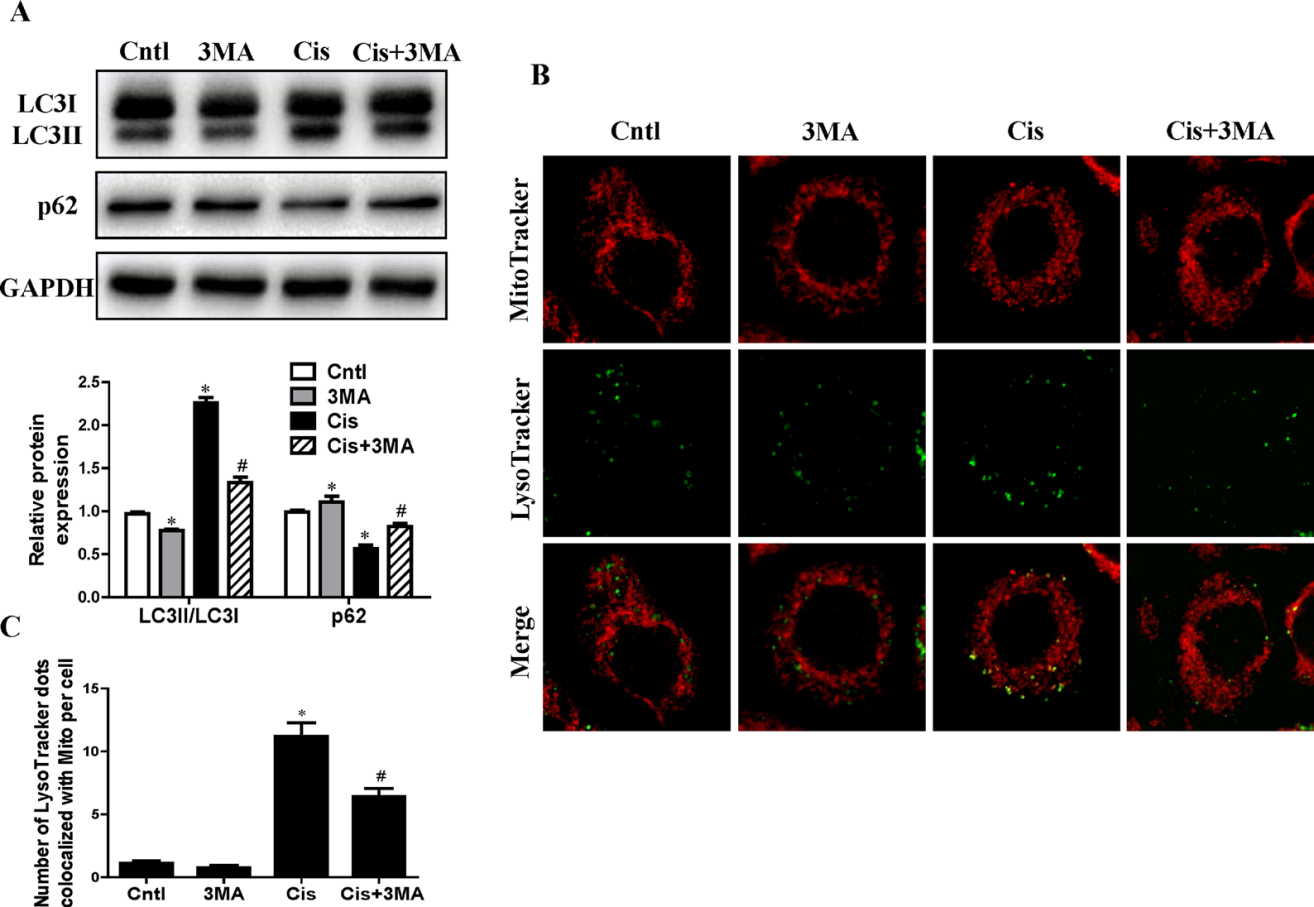
Effect of autophagy inhibitor on cisplatin-induced mitophagy HK2 cells were pre-treated with 3MA (5 mM) for 1 h, then incubated with cisplatin (10 uM) for further 12 h. (**A**) Western blotting analysis for LC3 conversion and p62. Upper: representative immunoblots. Lower: densitometric analysis. (**B**) Representative images of colocalization of lysosomes and mitochondria in HK2 cells. Mitophagy were detected by using MitoTracker Red and LysoTracker green staining. (**C**) Quantification of the number of LysoTracker-positive dots colocalized with MitoTracker in cells. More than 60 cells were counted for each group (*n* = 3). Data are expressed as the means ± SEM (*n* = 6). *P < 0.01 versus control. ^#^*P* < 0.01 versus cisplatin treatment group. Cntl, control group, Cis, cisplatin group, Cis+3MA, cisplatin+3MA group.

To determine the role of mitophagy in cisplatin treatment, the autophagy inhibitor 3-methyladenine (3-MA) was added to HK2 cells. 3-MA can inhibit autophagy due to suppression of class III phosphatidylinositol 3-kinase which is essential for the initiation of the early stages of autophagy [14]. As shown in Figure 2A, 3-MA inhibited cisplatin induced the conversion of LC3-I to LC3-II and the decrease of p62 expression. To observe the mitophagy, HK2 cells were co-loaded with red-fluorescing MitoTracker and green-fluorescing LysoTracker. As illustrated in Figure 2B and 2C, cisplatin increased the numbers of dual-labeled structures in HK2 cells, which was blocked by 3-MA pretreatment. As expected, these results indicated that cisplatin-induced autophagy and mitophagy were inhibited by 3-MA.

**Figure 3:**
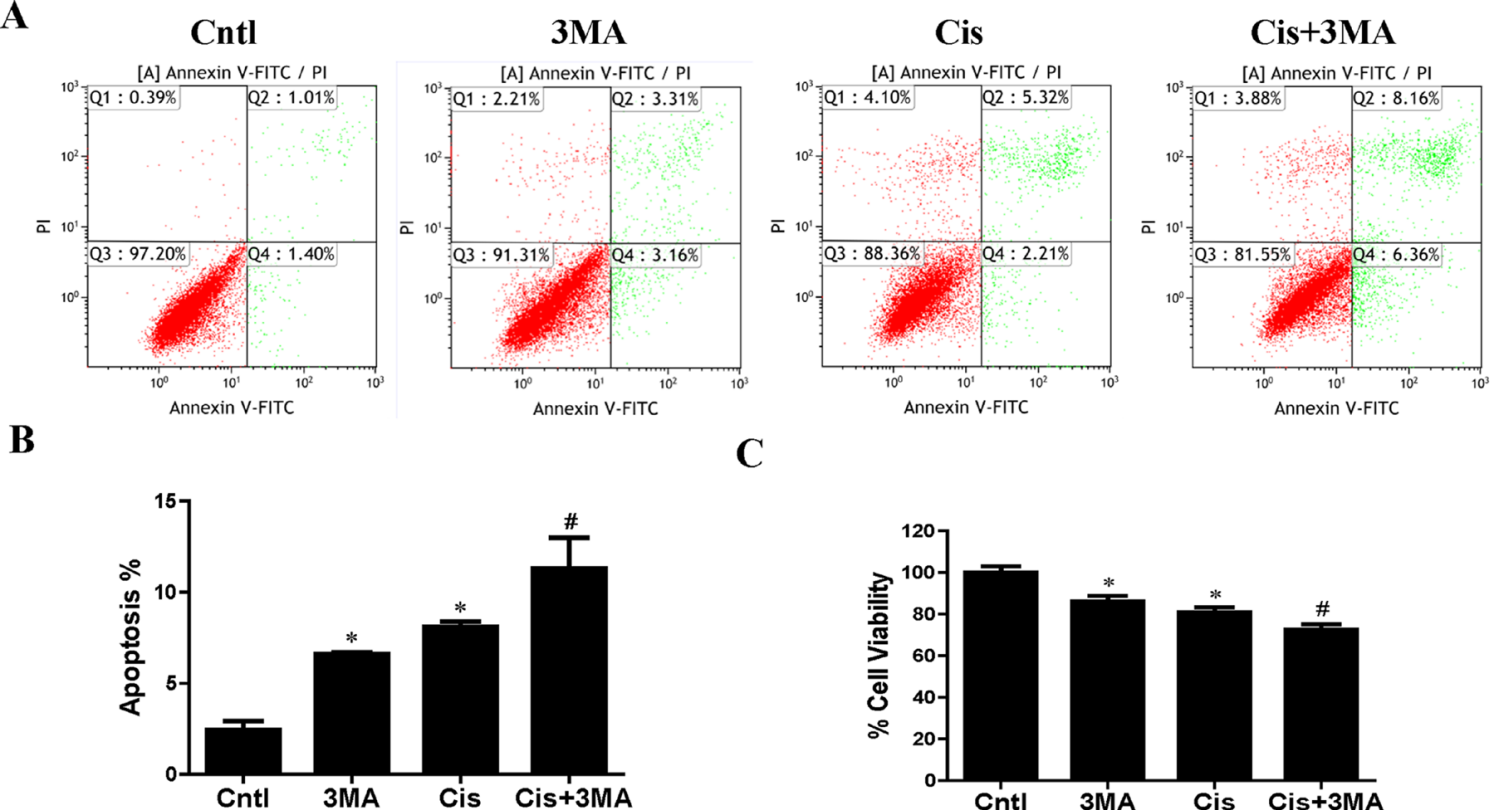
Effect of autophagy inhibitor on cisplatin-induced HK2 cells injury HK2 cells were pre-treated with 3MA (5 mM) for 1 h, then incubated with cisplatin (10 uM) for further 12 h. (**A**) The effects of 3MA on cisplatin-induced apoptosis were determined by flow cytometry. (**B**) Quantitation analysis of Annexin V+PI- and Annexin V+PI+ HK2 cells by flow cytometry. (**C**) Cell viability was determined by CCK-8 assay. Cell viability was expressed as percentage of controls. Data are expressed as the means ± SEM (*n* = 6). *P < 0.01 versus control. ^#^*P* < 0.01 versus cisplatin treatment group. Cntl, control group, Cis, cisplatin group, Cis+3MA, cisplatin+3MA group.

### Autophagy inhibitor accelerated cisplatin-induced HK2 cells injury

Next, we investigated the effects of autophagy/mitophagy inhibition on HK2 cells injury via 3-MA pretreatment. As shown in Figure 3A and 3B, 3C-MA notably enhanced cisplatin-induced apoptosis in HK2 cells. Also, CCK-8 assay revealed that treatment with 3-MA significantly increased cisplatin-induced cytotoxicity in HK2 cells (Figure 3C). These studies suggested that the induction of autophagy/mitophagy provided a prosurvival role during cisplatin-induced injury to cultured HK2 cells.

### Autophagy inhibitor accelerated cisplatin-induced mitochondrial dysfunction in HK2 cells

**Figure 4:**
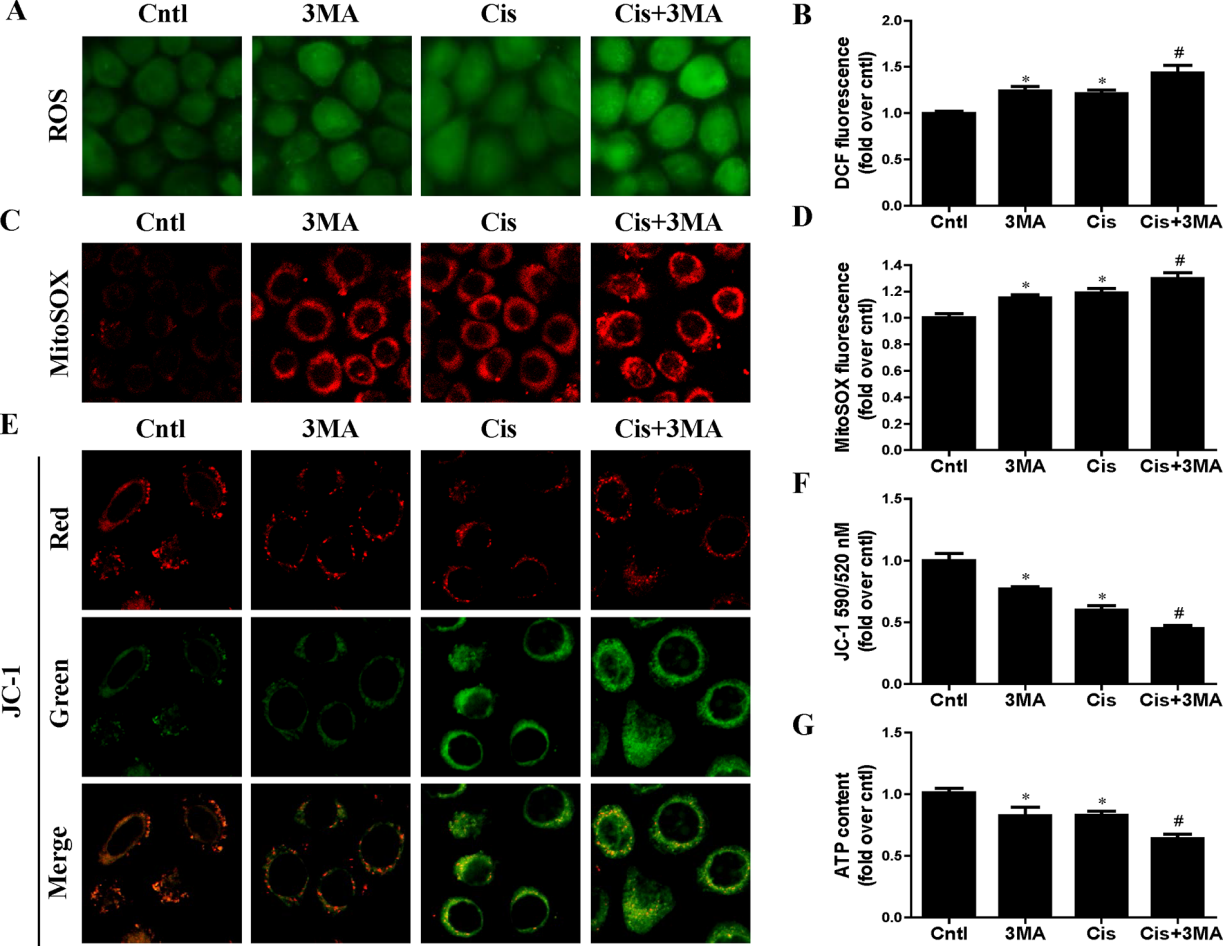
Effect of autophagy inhibitor on cisplatin-induced mitochondrial dysfunction HK2 cells were pre-treated with 3MA (5 mM) for 1 h, then incubated with cisplatin (10 uM) for further 12 h. (**A**) Representative images of HK2 cells stained with DCFDA (×200). (**B**) The DCF fluorescence intensities were analyzed by fluorescence microplate reader. (**C**) Representative images of HK2 cells stained with MitoSOX (×200). (**D**) Quantitation of MitoSOX by flow cytometry. (**E**) Representative images of HK2 cells stained with JC-1 (×200). (**F**) Mitochondrial membrane potential was detected by fluorescence microplate reader. (**G**) ATP contents were detected as described in Materials and Methods. Data are expressed as the means ± SEM (*n* = 6). *P < 0.01 versus control. ^#^*P* < 0.01 versus cisplatin treatment group. Cntl, control group, Cis, cisplatin group, Cis+3MA, cisplatin+3MA group.

To detect the association between mitophagy induction and the mitochondrial function, indicators of mitochondrial dysfunction such as ROS production, mitochondrial membrane potential and ATP generation were performed. As shown in Figure 4A and 4B, 3-MA pretreatment enhanced cisplatin-induced cellular ROS levels. Consistently, 3-MA greatly enhanced cisplatin-induced mitochondrial ROS production (Figure 4C and 4D). In addition, 3-MA accelerated cisplatin-induced loss of mitochondrial membrane potential (Figure 4E and 4F). Also, 3-MA aggravated the inhibitory effect of cisplatin on ATP production (Figure 4G). Thus, these results suggest that the activated mitophagy protects HK2 cells exposure to cisplatin probably by regulating mitochondrial function.

### Autophagy inducer enhanced cisplatin-induced mitophagy in HK2 cells

**Figure 5:**
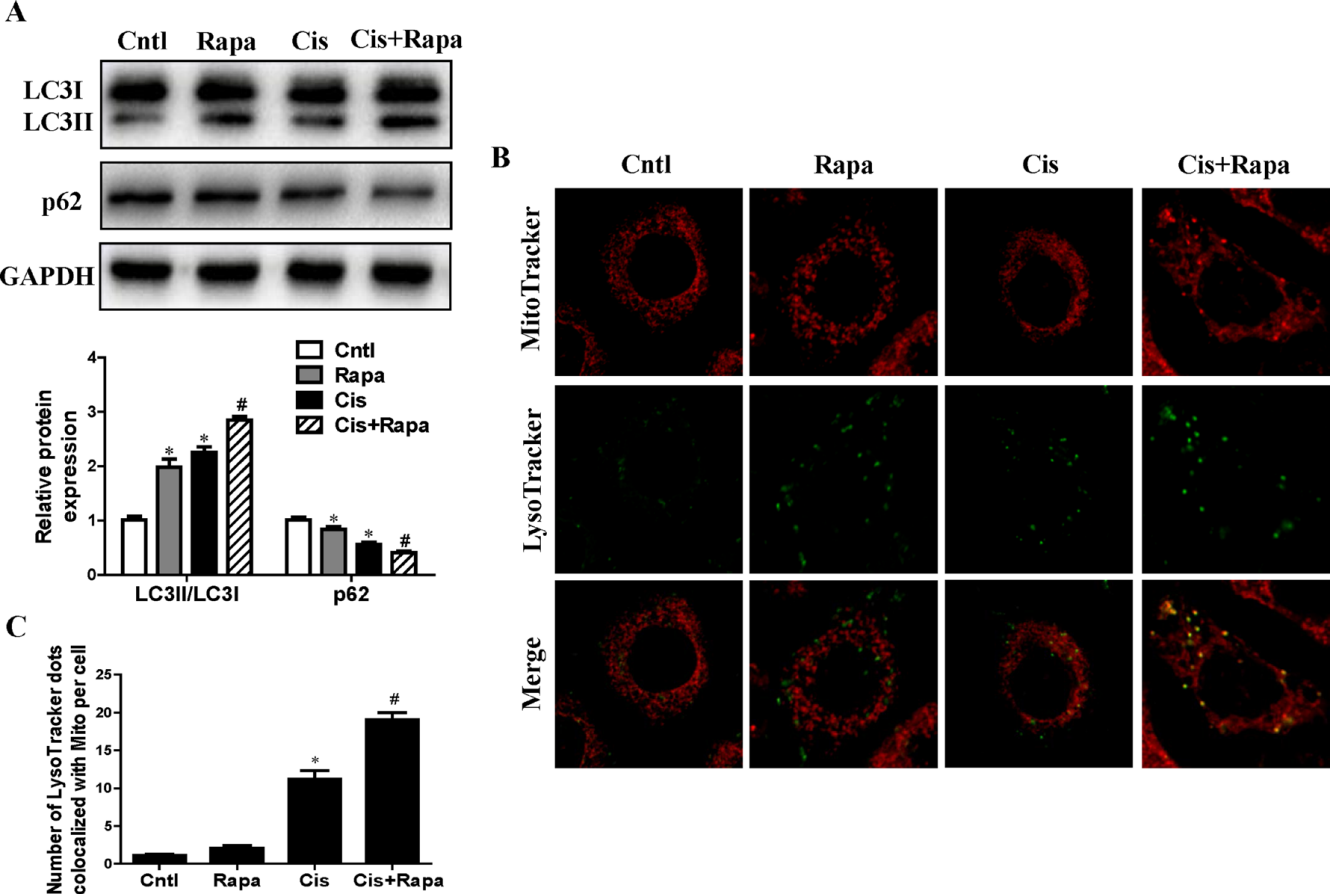
Effect of autophagy inducer on cisplatin-induced mitophagy HK2 cells were pre-treated with rapamycin (100 nM) for 1 h, and then incubated with cisplatin (10 uM) for further 12 h. (**A**) Western blotting analysis for LC3 conversion and p62. Upper: representative immunoblots. Lower: densitometric analysis. (**B**) Representative images of mitophagy detected by using MitoTracker Red and LysoTracker green staining. (**C**) Quantification of the number of LysoTracker-positive dots colocalized with MitoTracker in cells. More than 60 cells were counted for each group (*n* = 3). Data are expressed as the means ± SEM (*n* = 6). *P < 0.01 versus control. ^#^*P* < 0.01 versus cisplatin treatment group. Cntl, control group, Rapa, rapamycin group. Cis, cisplatin group, Cis+ Rapa, cisplatin+rapamycin group.

Given that mitophagy was cytoprotective during cisplatin injury of renal proximal tubular cells, we hypothesized that autophagy inducers can reduce HK2 cells injury by increasing mitophagy levels. As shown in Figure 5A, treatment with rapamycin significantly increased LC3-II conversion and decreased p62 expression. Furthermore, rapamycin remarkably induced mitophagy in HK2 cells, as indicated by the number of MitoTracker and LysoTracker positive dots (Figure 5B and 5C). These data were consistent with our hypothesis that rapamycin induced upregulation of mitophagy in HK2 cells.

### Autophagy inducer alleviated cisplatin-induced HK2 cells injury and mitochondrial dysfunction

**Figure 6:**
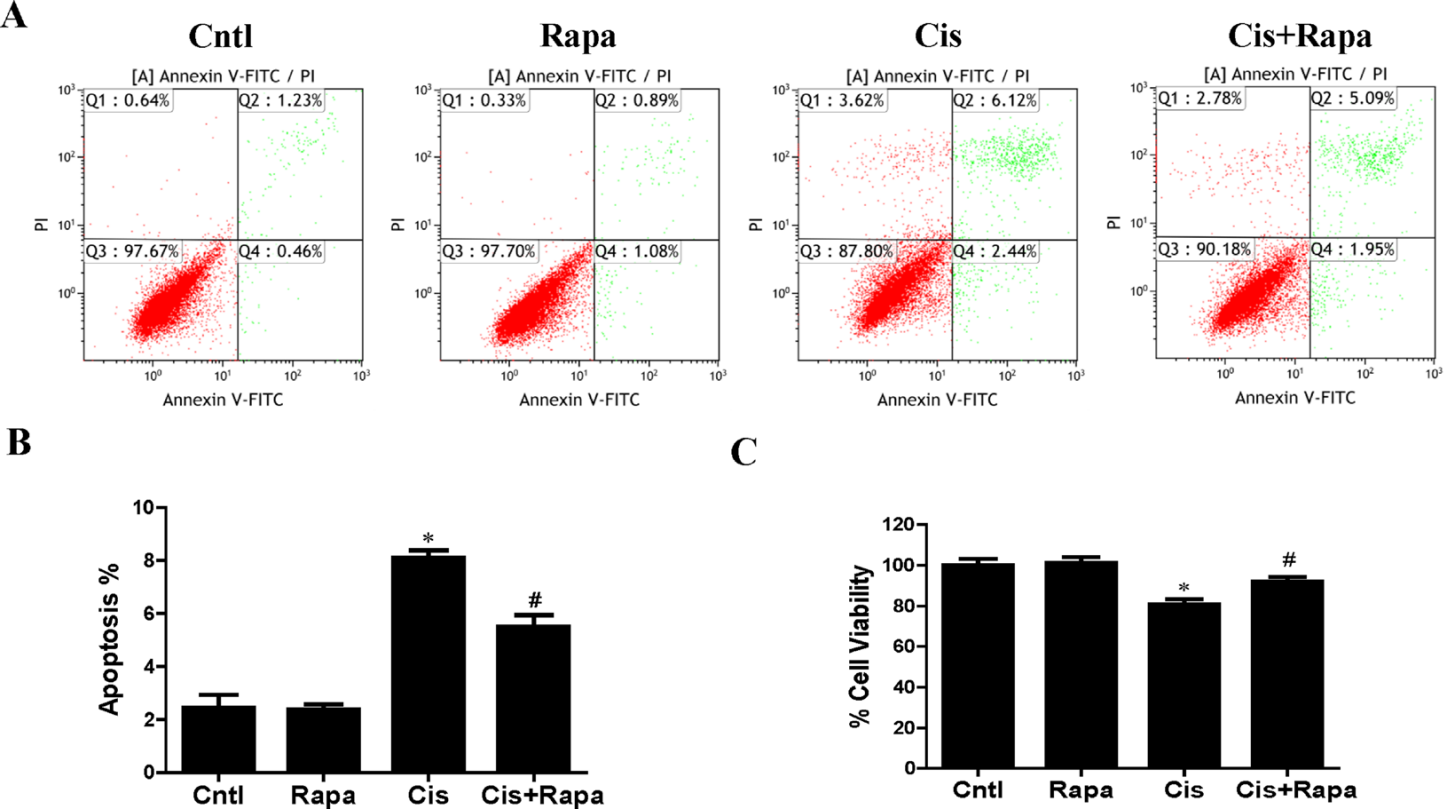
Effect of autophagy inducer on cisplatin-induced HK2 cells injury HK2 cells were pre-treated with rapamycin (100 nM) for 1 h, and then incubated with cisplatin (10 uM) for further 12 h. (**A**) The effects of rapamycin on cisplatin-induced apoptosis were determined by flow cytometry. (**B**) Quantitation analysis of apoptosis. (**C**) Cell viability was determined by CCK-8 assay. Data are expressed as the means ± SEM (*n* = 6). *P < 0.01 versus control. ^#^*P* < 0.01 versus cisplatin treatment group. Cntl, control group, Rapa, rapamycin group. Cis, cisplatin group, Cis+ Rapa, cisplatin+rapamycin group.

**Figure 7:**
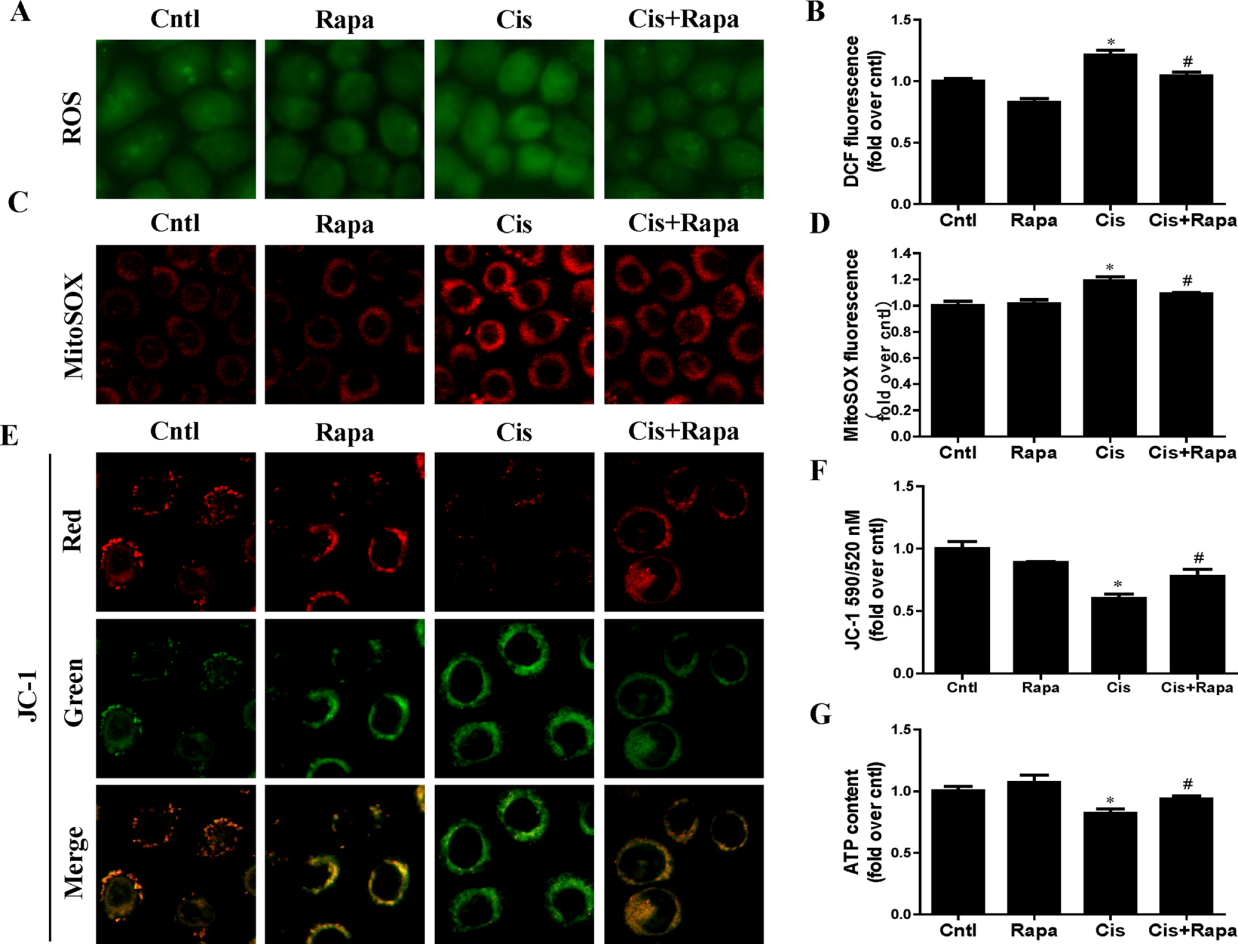
Effect of autophagy inducer on cisplatin-induced mitochondrial dysfunction HK2 cells were pre-treated with rapamycin (100 nM) for 1 h, and then incubated with cisplatin (10 uM) for further 12 h. (**A**) Representative images of HK2 cells stained with DCFDA (×200). (**B**) The DCF fluorescence intensities were analyzed by fluorescence microplate reader. (**C**) Representative images of HK2 cells stained with MitoSOX (×200). (**D**) Quantitation of MitoSOX by flow cytometry. (**E**) Representative images of HK2 cells stained with JC-1 (×200). (**F**) Mitochondrial membrane potential was detected by fluorescence microplate reader. (**G**) ATP contents. Data are expressed as the means ± SEM (*n* = 6). *P < 0.01 versus control. ^#^*P* < 0.01 versus cisplatin treatment group, Cntl, control group, Rapa, rapamycin group, Cis, cisplatin group, Cis+ Rapa, cisplatin+rapamycin group.

In contrast to autophagy/mitophagy deficiency, upregulation of mitophagy by rapamycin reduced cisplatin-induced cell apoptosis (Figure 6A and 6B) and cell death (Figure 6C). Impaired mitochondria are degraded by autophagy [15]. To confirm that the upregulation of autophagy could maintain mitochondrial function through the removal of the damaged mitochondrion in HK2 cells, we investigated the effect of rapamycin on cisplatin-induced mitochondrial dysfunction. Rapamycin treatment significantly reversed the increase of both total cellular and mitochondrial ROS levels after cisplatin treatment using DCFDA and MitoSOX Red, respectively (Figure 7A–7D). Rapamycin prevented cispaltin-induced mitochondrial membrane potential loss (Figure 7E and 7F). Similarly, rapamycin inhibited the decrease in ATP production induced by cisplatin (Figure 7G). These results indicated that the upregulating mitophagy levels by rapamycin inhibited cisplatin-induced HK2 cells injury and mitochondrial dysfunction.

### Suppression of mitochondrial fission by Drp1 knockdown inhibited cisplatin-induced mitophagy

**Figure 8:**
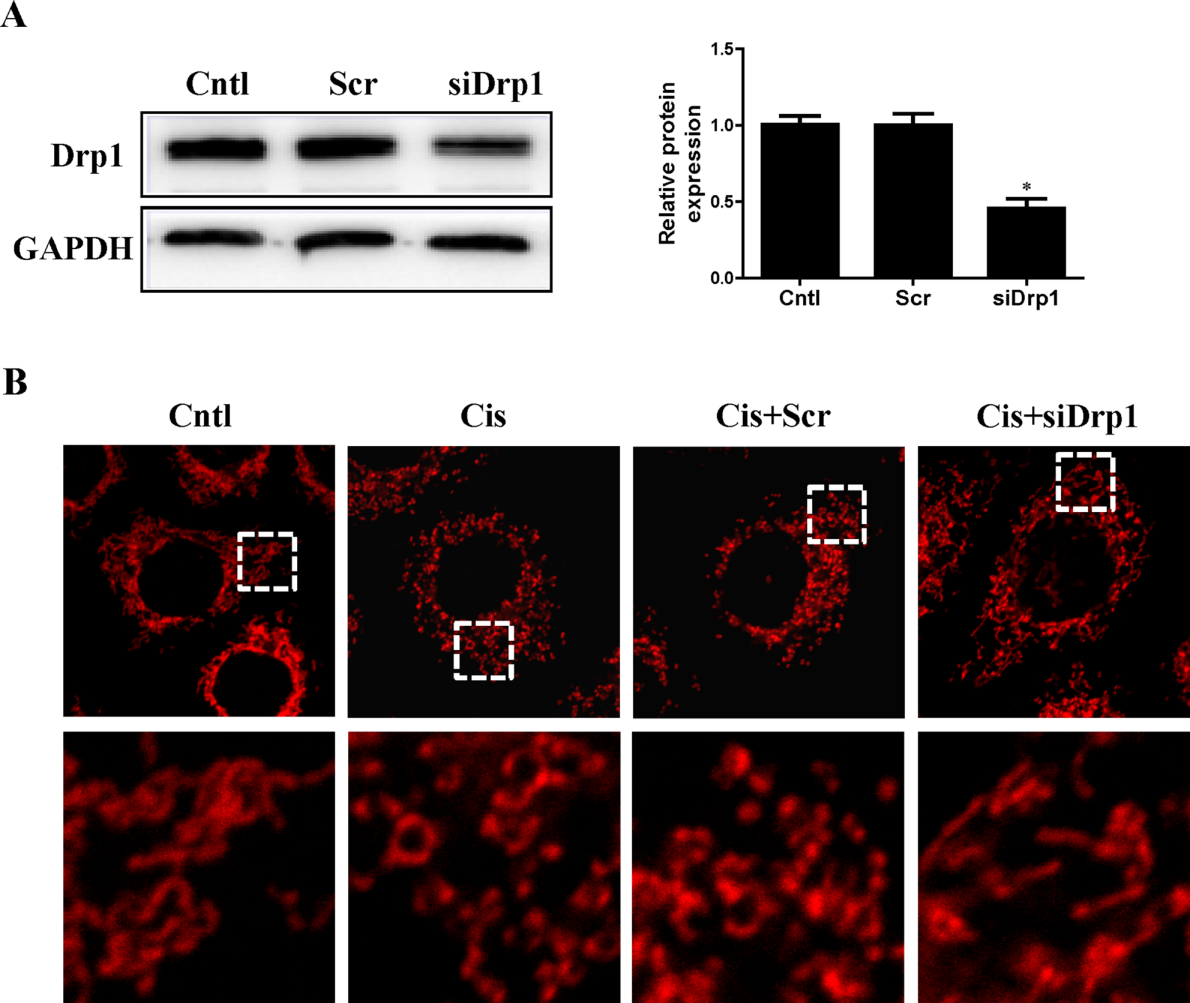
Effect of Drp1 silencing on cisplatin-induced mitochondrial fission HK2 cells were transfected with Drp1 siRNA or scrambled siRNA, with untreated cells used as the control (Cntl). (**A**) Drp1 protein expression was analyzed by western blotting. Right: representative immunoblots. Left: densitometric analysis. (**B**) Representative confocal images of mitochondrial morphologies in HK2 cells. HK2 cells were transfected with Drp1 siRNA or scrambled siRNA siRNA, then treated with cisplatin for 12 h. Mitochondria was stained by MitoTracker Red. Images in the box at higher magnification are shown in the below panels. Data are expressed as the means ± SEM (*n* = 6). *P < 0.01 versus control or scrambled siRNA group, Cntl, control group, Scr, scrambled siRNA group, siDrp1, Drp1 siRNA group, Cis, cisplatin group.

**Figure 9:**
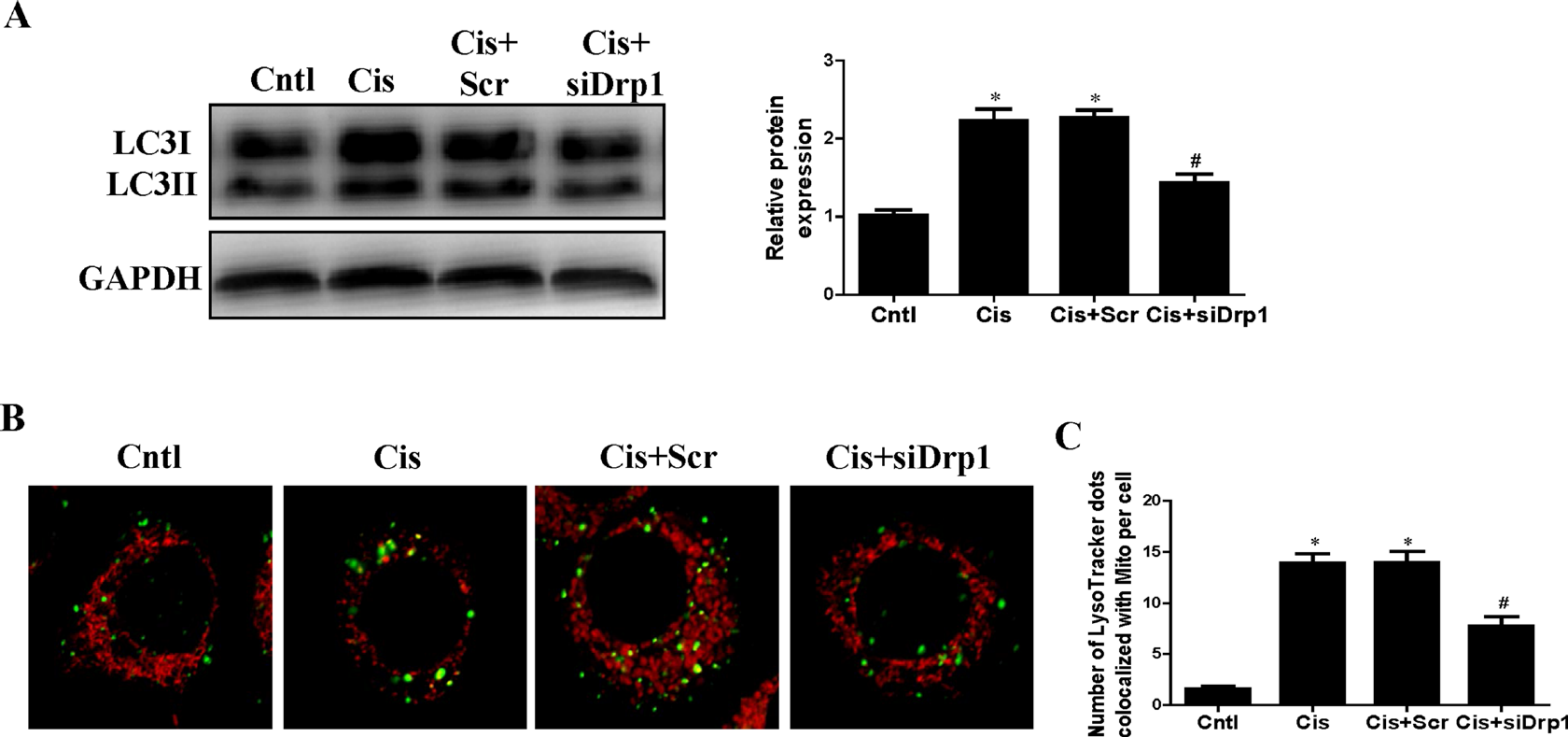
Effect of Drp1 siRNA on cisplatin-induced mitophagy HK2 cells were transfected with Drp1 siRNA for 24 h, then incubated with cisplatin (10 uM) for further 12 h. (**A**) Western blotting analysis for LC3 conversion. Right: representative immunoblots. Left: densitometric analysis. (**B**) Mitophagy were detected by using MitoTracker Red and LysoTracker green staining. (**C**) Quantification of the number of mitophagy. Data are expressed as the means ± SEM. *P < 0.01 versus control. ^#^*P* < 0.01 versus cisplatin treatment group, Cntl, control group, Cis, cisplatin group, Scr, scrambled siRNA group, siDrp1, Drp1 siRNA group.

Drp1 is known to be the master regulator of mitochondrial fission. To gain insight in the relevance of mitochondrial fission for cisplatin–induced mitophagy, the participation of this protein in mitophagy was assayed. As shown in Figure 8A, western blot analyses revealed a significant reduction in Drp1 protein expression in HK2 cells transfected with Drp1 siRNA as compared to the control. Cisplatin-induced mitochondrial fission was inhibited in cells transfected with Drp1 siRNA (Figure 8B). We next examined mitophagy after Drp1 knockdown. As shown in Figure 9A, Drp1 siRNA blocked the effect of cisplatin on LC3-II-to-LC3-I ratio. Similar results were observed in MitoTracker and LysoTracker co-staining study (Figure 9B and 9C). Thus, Drp1 mediated mitochondrial fission was essential for cisplatin-induced mitophagy.

### Suppression of mitochondrial fission inhibited cisplatin-induced HK2 cells injury and mitochondrial dysfunction

**Figure 10:**
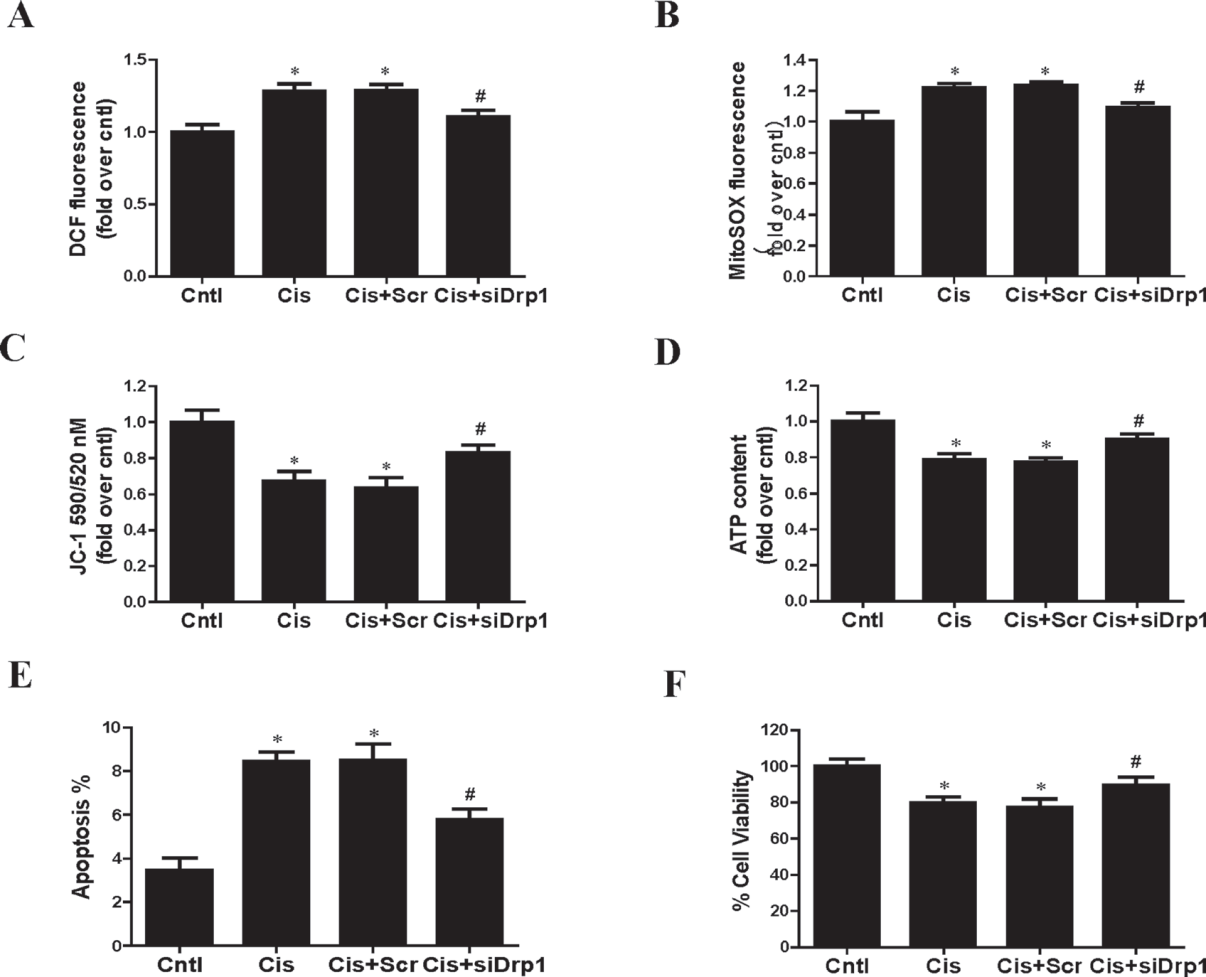
Effect of Drp1 siRNA on cisplatin-induced mitochondrial dysfunction and cell injury HK2 cells were transfected with Drp1 siRNA for 24 h, then incubated with cisplatin (10 uM) for further 12 h. (**A**) The DCF fluorescence intensities were analyzed by fluorescence microplate reader. (**B**) Quantitation of MitoSOX by flow cytometry. (**C**) Mitochondrial membrane potential was detected by fluorescence microplate reader. (**D**) ATP contents. (**E**) Quantitation analysis of apoptosis by flow cytometry. (**F**) Cell viability was determined by CCK-8 assay. Data are expressed as the means ± SEM (*n* = 6). *P < 0.01 versus control. ^#^*P* < 0.01 versus cisplatin treatment group. Cntl, control group, Cis, cisplatin group, Scr, scrambled siRNA group, siDrp1, Drp1 siRNA group.

As Drp1 regulated mitophagy, we further investigated the effects of Drp1 on cisplatin-induced mitochondrial dysfunction and HK2 cells injury. Knockdown of Drp1 abolished cisplatin-induced ROS generation using DCFDA staining (Figure 10A) and MitoSOX staining (Figure 10B). Consistently, knockdown of Drp1 prevented cisplatin-induced reduction in mitochondrial membrane potential (Figure 10C) and ATP production (Figure 10D). Moreover, Drp1 siRNA inhibited cisplatin-induced HK2 cells injury accessed by cell apoptosis (Figure 10E) and cell viability (Figure 10F). Therefore we concluded that suppression of mitochondrial fission by Drp1 knockdown protected against cisplatin-induced HK2 cells injury by inhibiting mitochondrial dysfunction.

## DISCUSSION

In this study, we demonstrated that mitophagy was induced in renal tubular cells during cisplatin treatment. Indeed by facilitating the clearance of impaired mitochondria this selective autophagy triggered by cisplatin displayed an additional protective function. Our data further showed that the blockage of Drp1, a core regulatory molecule for fission, abolished mitochondrial fission and mitophagy induced by cisplatin in HK2 cells.

Acute kidney injury (AKI), caused by sepsis, ischemia or nephrotoxic agents, is characterized by abrupt and reversible kidney dysfunction [16]. AKI is an increasingly common and potentially catastrophic complication in hospitalized patients [17]. AKI leads to acute cell death and necrosis of renal tubular epithelial cells [18]. Moreover, AKI is an important predictor in the development and progression of chronic kidney disease [19]. The commonly used as first line chemotherapy drug cisplatin accumulates preferentially in the renal tubular cells and is a frequent cause of drug-induced AKI [20]. Recently, cumulative evidences supported a cyto-protection role of autophagy in cisplatin-induced AKI [21]. Previous studies reported that the administration of autophagy inducer rapamycin attenuated cisplatin nephrotoxicity whereas inhibition of autophagy exacerbated the cisplatin AKI model in mice [22]. Consistently, our results also found that 3-methyladenine, a pharmacological inhibitor of autophagy, blocked autophagic flux and enhanced cisplatin-induced renal proximal tubular cells injury. In contrast, rapamycin activated autophagy and protected against injury in renal proximal tubular cells exposed to cisplatin. However, the function of autophagy in different causes of AKI is still controversial. In proximal tubule cells subject to oxidant injury via hydrogen peroxide or hypoxia, an increase in autophagy mediated an increase in cell death [23].

Mitochondrial dysfunction is a major contributor to tubular cell injury observed in the initiation and progression of AKI [24]. Mitochondria are a significant source of ROS. The elevated ROS level within mitochondria is deleterious to the cell due to their ability to induce lipid peroxidation, protein oxidation and DNA damage [25]. Indeed, mitochondria also the major targets of oxidative stress, whereas ROS represents the trigger for several mitochondrial dysfunctions [26]. Furthermore, the mitochondrial inner membrane is vital for ATP production via the electron-transport chain. It was reported that cisplatin decreased mitochondrial membrane potential, which resulted in the collapse of energy generation and ATP depletion, sensitizing HK2 cells to apoptosis [3]. In agreement with these findings, our data showed that cisplatin induced production of ROS and reduction of mitochondrial membrane potential and ATP content. Timely removal of damaged mitochondria is thus critical for cellular homeostasis and function. Mitophagy is the selective degradation of mitochondria by autophagy, and it plays an important role in the quality control of mitochondria [27]. Although mitophagy are considered of particular importance in protecting against AKI, evidence that activation of mitophagy improved mitochondrial function is lacking. Our results demonstrated that mitophagy was induced after cisplatin treatment, which was suppressed by autophagy inhibitor and enhanced by autophagy activator, respectively. Mitophagy served a protective role in limiting mitochondrial dysfunction and cell injury.

Both mitochondrial dynamics and mitophagy are integral components of the mitochondrial quality control machinery [28]. It has been identified that normally elongated mitochondria in the proximal tubule rapidly fragment in response to cisplatin [29]. Drp1 is the master regulator of mitochondrial morphology [30]. Drp1 mRNA is highly expressed in the human kidney and therefore may play a crucial role in the pathophysiology of mitochondrial-targeted injuries to kidney [24]. Previous study showed that a pharmacological inhibitor of Drp1 can ameliorate mitochondrial fission during renal ischemia-reperfusion and cisplatin nephrotoxicity *in vivo* [31]. Similar renoprotective effects were observed in rhabdomyolysis-induced AKI model, in which suppression of Drp1 accumulation in mitochondria favors the maintenance of mitochondrial function and reduces the apoptosis of tubular cells [32]. In the present study, suppression of Drp1by siRNA blocked cisplatin-induced mitochondrial fission, mitophagy, mitochondrial dysfunction and cell injury.

Mitochondrial dynamics and mitophagy are closely related. Mitochondrial fusion is important for inheritance and maintenance of mitochondrial DNA, whereas severely damaged mitochondria are separated from the mitochondrial network by fission and subsequently degraded by mitophagy [33]. Remarkably, inhibition of fission decreases mitophagy, and arrest of autophagy leads to the accumulation of mitochondria with low membrane potential [34]. Our data are in agreement with studies showing that mitochondrial fission must occur prior to mitophagy. Furthermore, mitochondrial membrane depolarization has been shown to precede the induction of mitophagy by nutrient deprivation in hepatocytes [35]. Recently, PINK1/Parkin and BNIP3L/NIX pathways were found to promote degradation of mitochondria by triggering the loss of mitochondrial membrane potential [36], but how these potential signals are integrated is not clear. The precise molecular mechanism by which damaged mitochondria are recognized by mitophagy in proximal tubular cells needs to be elucidated in the future.

In conclusion, we demonstrated here that mitophagy is protective in cisplatin-induced tubular cell injury via ameliorating mitochondrial impairment. Mitophagy is probably mediated by Drp1-dependent mitochondrial fission in tubular cells when treated with cisplatin. Therefore, targeting mitophagy may be a potential therapeutic strategy in cisplatin-induced nephrotoxicity.

## MATERIALS AND METHODS

### Reagents and antibodies

Cisplatin, 3-methyladenine (3MA) and Rapamycin (Rapa) were obtained from Sigma-Aldrich (St. Louis, MO). Anti-p62 antibody was obtained from Abcam (Cambridge, MA). Anti-LC3 antibody was purchased from Sigma-Aldrich (St. Louis, MO). Anti-Drp1 antibody was purchased from Santa Cruz (Dallas, TX). Anti-GAPDH antibody was purchased from Cell Signaling Technology (Beverly, MA).

### Cell culture

Human renal proximal tubular cells (HK2) were purchased from the American Type Culture Collection (Manassas, VA) and maintained in DMEM/F12 media supplemented with 10% FBS and antibiotics (100 U/ml penicillin G, 100 μg/mL streptomycin). All cells were grown at 37°C in 5% CO2 in a humidified incubator. Before cisplatin treatments, cells were serum starved for 16 to 20 hours.

### RNA interference

Small interfering RNA (siRNA) against human Drp1 and control scrambled siRNA were purchased from Santa Cruz Biotechnology (Santa Cruz, CA). HK2 cells were transiently transfected with 100 nM siRNA constructs using Lipofectamine 2000 (Invitrogen, Paisley, UK) according to the manufacturer's recommendations. Efficiency of knockdown was performed through Western blot analysis.

### Western blotting

Western blot analysis was conducted as described previously. In brief, the harvested cells were lysed on ice for 10 min using lysis buffer. Thirty micrograms of proteins were subjected to 12% SDS-PAGE and transferred to nitrocellulose membranes. After being blocked for 2 h in Tris-buffered saline with 0.1% Tween-20 (TBST) and 3% BSA, the membranes were probed with appropriate primary antibodies overnight at 4°C followed by incubation with peroxidase conjugated secondary antibodies. The blots were visualized with Amersham™ ECL™ Detection Systems (Amersham, Buckinghamshire, UK). Densitometric analysis was performed using Quantity One Software (Bio-Rad, Hercules, CA, USA).

### Detection of mitophagy

Mitophagy was determined by the co-localization of mitochondria with lysosome. The cells were co-loaded with 200 nM MitoTracker Red (Molecular Probes, Eugene, OR) and 1 μM LysoTracker Green (Molecular Probes, Eugene, OR) for 20 min. Images were acquired using a laser scanning confocal microscope (Carl Zeiss, Oberkochen, Germany). The numbers of mitochondria co-localized with lysosome were quantified.

### Transmission electronic microscope (TEM)

After treatment, HK2 cells were fixed with 2.5% glutaraldehyde/1.2% acrolein in fixative buffer (0.1 mol/l cacodylate, 0.1 mol/l sucrose, pH 7.4) and 1% osmium tetroxide. Ultrathin sections were stained with uranyl acetate for examination under an electron microscope (JEOL JEM-1010, Tokyo, Japan).

### Determination of intracellular ROS

The cellular ROS level was measured by a DCFDA fluorescent dye (Molecular Probes, Eugene, OR). Mitochondrial superoxide anion generation was detected by using MitoSOX (Molecular Probes, Eugene, OR) as described previously [37]. Briefly, after treatment, HK2 cells were incubated with 10 μM DCFDA for 30 min or 5 uM MitoSOX for 30 min. Images of fluorescently labeled cells were captured by a standard confocal laser scanning microscope (Carl Zeiss, Germany). The DCF fluorescence intensity was analyzed on BMG LABTECH FLUOstar OPTIMA Microplate Reader (Ortenberg, Germany) and MitoSOX fluorescence levels were analyzed by flow cytometry.

### Detection of mitochondrial membrane potential and mitochondrial morphology

Mitochondrial membrane potential was assessed by staining the cells with the JC-1 fluorescence dye (Molecular Probe, Eugene, OR). Briefly, treated cells were washed twice with PBS and JC-1 was added to each sample with a final concentration of 300 nM for 20 minutes. Fluorescence intensities were recorded by a FLUOstar Optima reader (BMG LABTECH, Ortenberg, Germany) and data was analyzed as described previously [38]. Mitochondrial membrane potential was expressed as the ratio of red to green fluorescence areas. To visualize the mitochondria, cells were stained with 200 nM MitoTracker Red (Molecular Probes, Eugene, OR) for 30 min before confocal microscopic analysis.

### Detection of intracellular ATP

ATP concentration was quantitatively determined using an ATP determination kit (Beyotime, Nanjing, China) according to the manufacturer's protocol. ATP content was expressed as nmol/mg protein.

### Cell viability and cell apoptosis

The cell viability was measured by using CCK-8 assay (Beyotime, Shanghai, China) according to the manufacturer's instructions. Cell viability was expressed as a percentage of the control cells. Apoptosis in cells was quantified by flow cytometry using an AnnexinV-fluorescein isothiocyanate-PI (AnnexinV-FITC–PI) double staining kit (BD Biosciences, San Diego, CA) according to the manufacturer's instructions. Both Annexin V^+^/PI^−^ cells representing early-apoptotic cells and Annexin V^+^/PI^+^ mostly representing late-apoptotic cells were considered as apoptotic cells.

### Statistical analysis

All data were presented as mean ± standard error (SE). Statistical analysis used one-way analysis of variance followed by Bonferroni's test with the SPSS 13.0 statistical software (SPSS, Chicago, IL). All results were considered significant at *p* < 0.05.
